# Phosphomimetic Thrombospondin-1 Modulates Integrin β1-FAK Signaling and Vascular Cell Functions

**DOI:** 10.3390/biom16010084

**Published:** 2026-01-04

**Authors:** Assala Raya, Bálint Bécsi, Anita Boratkó

**Affiliations:** Department of Medical Chemistry, Faculty of Medicine, University of Debrecen, Egyetem tér 1, 4032 Debrecen, Hungary; assala.raya@med.unideb.hu (A.R.); bbalint@med.unideb.hu (B.B.)

**Keywords:** endothelial cells, smooth muscle cells, thrombospondin 1, cell migration, cell signaling, atherosclerosis

## Abstract

Thrombospondin-1 (TSP1) is a multifunctional glycoprotein that plays a crucial role in angiogenesis and vascular remodeling. Ser93 of TSP1 has recently been identified as a novel phosphorylation site, influencing angiogenic properties; however, the underlying signaling mechanism remains unclear. Here, we investigated the functional impact of Ser93 phosphorylation using phosphomimetic (TSP1^S93D^) and phosphonull (TSP1^S93A^) mutants. Endothelial cell (EC) migration was analyzed using scratch assay and electric cell-substrate impedance sensing. Activation of key pathways (Akt, p38, ERK, and FAK) was analyzed by immunoblotting. TSP1 secretion was quantified by ELISA. Downstream effects on smooth muscle cells were examined by Western blot using conditioned media of endothelial cells. Expression of TSP1^S93D^ significantly impaired endothelial migration and wound closure, associated with reduced phosphorylation of FAK and paxillin. Upstream of FAK signaling, TSP1^S93D^ showed enhanced binding to integrin β1 and promoted its clustering. In contrast, TSP1^S93D^ stimulated smooth muscle cell proliferation, migration, cytoskeletal remodeling, and phenotypic switching toward a synthetic, pro-inflammatory state characterized by elevated marker protein expression. Together, these findings demonstrate that the impaired angiogenic properties induced by TSP1^S93D^ result from the modulation of integrin β1-FAK pathways in ECs, suppressing endothelial motility while promoting smooth muscle activation, suggesting a role in early vascular remodeling and inflammation.

## 1. Introduction

The proper functioning of blood vessels depends heavily on the coordinated activity of several cell types, which together regulate blood circulation, coagulation, blood pressure, and processes like inflammation and angiogenesis [1,2,3]. The first structural barrier is formed by endothelial cells (ECs), which line the inner surface of the vessels and act as a semi-permeable barrier between the blood and surrounding tissue. Surrounding the endothelium are vascular smooth muscle cells (VSMCs), which primarily communicate with ECs through paracrine signaling. This dynamic and reversible interaction supports proper vascular formation and enables adaptive changes in response to physiological needs, even affecting gene and protein expression [4]. These cellular dialogues play a central role in determining vascular architecture and function [5]. The extracellular matrix (ECM), besides providing structural support, directly affects EC behavior, such as movement, adhesion, spreading, and sensitivity to external signals under both normal and pathological conditions [6]. Among the many ECM-associated proteins, the thrombospondin (TSP) family consists of five calcium-binding extracellular glycoproteins, each capable of interacting with various ECM components in both transient and sustained ways [7,8]. Thrombospondin-1 (TSP1) is the most well-known member of the TSP family. Its regulatory role in cell migration, proliferation, or even apoptosis has been shown in connection with inflammation, wound healing, angiogenesis, or tumor progression [8,9]. Vascular cells are among the many cell types that express and release TSP1 upon activation, contributing to the modulation of cell–cell and cell–matrix interactions as well as the regulation of cellular activity. Structurally, TSP1 is characterized by several TSP-type repeats (TSRs), namely, type 1, 2, and 3; therefore, it is a member of the TSR supergene family [10]. It also has distinct N- and C-terminal globular domains and a procollagen-like (PC) region or, by another name, the von Willebrand type C (vWC) domain. TSP1 exerts its biological effects through interactions with various cell surface receptors, such as syndecans, low-density lipoprotein receptor-related protein 1 (LRP1), CD36, integrins, and CD47 [11].

TSP1 was first characterized as an endogenous angiogenesis inhibitor [12]; however, the specific functional or structural domain involved determines whether TSP1 has pro-angiogenic or anti-angiogenic effects in different pathological settings [13,14]. TSP1 binds CD47 with high affinity, making CD47 its primary receptor in vivo [15]. The interaction triggers multiple downstream signals in endothelial cells. Engagement of CD47 by TSP1 inhibits nitric oxide signaling, stimulates reactive oxygen species production, and suppresses vascular endothelial growth factor receptor 2 (VEGFR2)-mediated angiogenesis [16,17]. Its inhibitory effect on NO signaling occurs downstream of cyclic guanosine monophosphate (cGMP), altering endothelial cell responses, including ERK activity, by preventing the NO-induced increase in extracellular signal-regulated kinase (ERK) phosphorylation [18,19]. Besides the well-characterized TSP1-CD47 axis, another extensively studied pathway is the TSP1-CD36 interaction. It can activate Toll-like receptor 4 (TLR4) signaling, and as a downstream target, nuclear factor kappa B (NF-κB) promotes pro-inflammatory cytokine production [20]. CD36 also facilitates fatty acid uptake [21], and TSP1-CD36 engagement inhibits migration and VEGFR2-mediated signaling [22]. TSP1 can suppress angiogenesis and promote apoptosis by activating CD36 via the p38 and caspase-dependent pathways [22,23]. TSP1 regulates EC adhesion and migration via the interactions with integrins like α3β1, α4β1, α6β1, and α9β1 through its N-terminal domain [24], and several studies have shown the involvement of TSP1 in multiple pathways during cell migration [25,26,27,28,29]. TSP1 secretion in EC depends on both calcium signaling and post-translational modifications, particularly the heavy glycosylation of TSP1 [30,31]. By orchestrating supramolecular complexes that dynamically change in composition over time, TSP1 helps regulate EC responses to environmental stimuli. In addition to its inhibitory effects on ECs, TSP1 exerts opposing effects on VSMCs. While it suppresses EC migration and proliferation and prevents the spontaneous formation of angiogenic tube-like structures [32], TSP1 promotes VSMC migration and proliferation [33]. Studies using TSP1 antibodies have demonstrated that TSP1 is essential for VSMC proliferation, making it a positive regulator of vascular smooth muscle development [34]. In cases of mild vascular injury, the absence of TSP1 delays and disrupts arterial SMC activation, leading to reduced neointima formation [35]. This highlights the essential role of TSP1 in vascular repair and remodeling. The effect of TSP1 on VSMC behavior like proliferation or phenotype switching is crucial in vascular remodeling. It contributes to the evolution of vascular diseases and the progression of atherosclerosis [36,37].

Beyond its interactions with various receptors and proteins, TSP1 undergoes post-translational modifications at multiple sites within its sequence, which significantly impact its activity and binding properties [38,39]. Recently, we identified that TSP1 is regulated by reversible phosphorylation at the Ser93 side chain [40]. In 3D spheroid models, expression of the phosphomimetic TSP1 mutant (TSP1^S93D^) led to less stable spheroids and reduced capillary sprouting compared with wild-type TSP1 (TSP1^WT^) or a phosphonull mutant (TSP1^S93A^). Short peptides derived from TSP1 exert potent anti-angiogenic effects by mimicking key inhibitory sequences of the full-length protein [41]. Based on these properties, synthetic TSP1 mimetic peptides, such as ABT-510, have been evaluated in clinical settings as regulators of pathological angiogenesis [42]. The identification of Ser93 as a novel phosphorylation site in TSP1 introduces an additional level of regulatory complexity and suggests the potential for developing new peptide-based modulators targeting this region. Elucidating the downstream mechanism associated with this modification represents a necessary step toward exploiting this site for therapeutic peptide development. The present study investigates how TSP1 Ser93 side chain phosphomutations affect EC migration, adhesion signaling, and communication with VSMCs.

## 2. Materials and Methods

### 2.1. Materials

Materials were acquired from the following sources: Phosphate-Buffered Saline tablet (PBS, P4417), Bovine Serum Albumin (BSA, A9647), Dimethyl sulfoxide (DMSO; D8418), Tween 20 (P7949), 2-Mercaptoethanol (M3148), Triton X-100 (T8787), Paraformaldehyde (PFA, P6148), isopropyl β-D-1-thiogalactopyranoside (IPTG, I5502), N,N,N′,N′-Tetramethyl-ethylenediamine (TEMED, T7024), and methanol (1.06007.2500) from Sigma (St. Louis, MO, USA); Nuclease-free water (E478), Tris (hydroxymethyl) aminomethane (TRIS, Trometamol, 33621.260), Sodium chloride (NaCl, 27788.29), acetic acid glacial (20104.334), and ethanol (20821.296) from VWR International (Radnor, PA, USA); Acrylamide/Bisacrylamide Solution 40% (J60868.AP) and sodium dodecyl sulfate (SDS, 419530010) from Thermo Scientific, Inc. (Vantaa, Finland); Glycine (G0709.1000) from Duchefa Biochemie (Haarlem, The Netherlands); Protino^®^ Glutathione Agarose 4B (11962462) from Macherey-Nagel (Düren, Germany); Protein G Sepharose (17-0618-01) from GE Healthcare Bio-Science (Uppsala, Sweden), Ammonium persulfate (AMPER, 13375) from SERVA Electrophoresis GmbH (Heidelberg, Germany); Complete Mini protease inhibitor cocktail (11836153001) from Roche Diagnostics (Basel, Switzerland). Reagents for cell culture were obtained as follows: Minimum Essential Medium with Earle’s Salts and L-Glutamine (MEM-A), Dulbecco’s Modified Eagle Medium with L-Glutamine and Sodium Pyruvate (DMEM-HPA), Fetal Bovine Serum collected in South America (FBS-12A) from Capricorn Scientific GmbH (Ebsdorfergrund, Germany); Non-essential Amino Acid mixture (13-114E) and Na Pyruvate (BE13-115E) were acquired from Lonza Group AG (Basel, Switzerland); Trypsin/EDTA (LM-T1706/500) was obtained from Biosera (Nuaillé, France). All chemicals used were of molecular biology or analytical grade.

### 2.2. Cell Cultures

Bovine pulmonary artery endothelial cells (BPAECs) (culture line CCL 209) were obtained from the American Type Tissue Culture Collection (ATCC), Rockville, MD, USA) and subsequently used at passages 17–20, as described in [43]. Mouse vascular aortic smooth muscle (MOVAS, culture line CRL-2797) was obtained from ATCC and used at passages 10–20. Cells were maintained in DMEM supplemented with 10% (*v*/*v*) FBS at 37 °C in a humidified atmosphere containing 5% CO_2_ and 95% air.

### 2.3. SDS-PAGE and Western Blotting

Polyacrylamide gels were prepared using the Laemmli method. Gels consisted of a 4% stacking gel and either a 10% or 12% separating gel, depending on the experimental requirement. Electrophoresis was performed in Tris-Glycine running buffer (25 mM Tris, 192 mM glycine, 0.1% SDS, pH~8.3). Following SDS-PAGE, proteins were transferred to a 0.45 μm nitrocellulose membrane (#88018, Thermo Scientific, Rockford, IL, USA), as in [44]. The utilized primary and secondary antibodies with their respective dilutions are listed in Table 1. For lectin immunoblotting, blocked membranes were incubated with biotinylated lectin (L5142, Sigma Aldrich, St. Louis, MO, USA) diluted in 3% BSA in TBS (Tris-buffered saline: 20mM Tris-HCl; 150mM NaCl, pH 7.5) overnight at 4 °C. After washing with TBST (TBS, 0.1% Tween-20), membranes were incubated with horseradish peroxidase (HRP)-conjugated streptavidin (OR03L, Sigma Aldrich, St. Louis, MO, USA) for 1 h at room temperature. Chemiluminescent signals were detected and imaged using a Bio-Rad ChemiDoc Touch imaging system (Bio-Rad Laboratories, Hercules, CA, USA). The original, uncropped Western blot images corresponding to the figures in this manuscript are provided in the Supplementary Material.

### 2.4. Transfection and Scratch Assay

BPAECs were transfected with pcDNA3.1 c-myc/His-A TSP1 (TSP1^WT^, TSP1^S93A^, and TSP1^S93D^) constructs created and successfully applied earlier [40]. The transfection was completed using Lipofectamine 3000 reagent (Invitrogen Corporation, Carlsbad, CA, USA) according to the manufacturer’s protocol. Twenty-four hours post-transfection, a scratch assay was performed on a confluent monolayer of cells, as in [40]. After scratching in PBS, the buffer was removed and replaced with conditioned media. Images were acquired at 0, 8, and 24 h, and wound closure was quantified using ImageJ software 1.54g.

### 2.5. Electric Cell-Substrate Impedance Sensing (ECIS) Measurements

To assess cell migration, ECIS model Zθ (Applied BioPhysics Inc. (Troy, NY, USA)) was used [45,46]. Briefly, BPAECs were seeded onto 8W10E arrays and transfected with TSP1 constructs. For MOVAS cells, complete conditioned media from TSP1-expressing ECs was added following seeding. The confluent monolayer of cells was electrically wounded (5 mA, 60 kHz, 30 s) 24 h post-transfection or treatment, and impedance changes were recorded for 10 h. Migration rate was calculated as previously mentioned in [47].

### 2.6. High Content Screening (HCS) Confocal Microscopy

BPAECs or MOVAS cells were seeded in CellCarrier Ultra 96-well microplates (Perkin Elmer, Waltham, MA, USA). To analyze morphological changes in MOVAS cells, cells were plated at 10% confluence with conditional media from transfected BPAECs containing an equal amount of recombinant TSP1 (300 ng/mL) [48]. Cells were fixed with 3.7% PFA in PBS for 10 min at 24 h post-treatment and then permeabilized with 0.5% Triton X-100 in PBS for 20 min and blocked with 2% BSA in PBS for 30 min at room temperature. ITGB1 antibody was diluted in blocking solution (1:100). Actin cytoskeleton was labeled using Texas-Red Phalloidin (T7471, Invitrogen, Eugene, OR, USA) at 1:300 dilution, and nuclei were stained with 1 μg/mL DAPI (4′,6-diamidino-2-phenylindole) (D9542, Sigma, St. Louis, MO, USA). Between each step, cells were rinsed three times with 1× PBS. Imaging was performed using Opera Phenix High-Content Analysis System (Perkin Elmer, Waltham, MA, USA) with a 63× water immersion objective. Image analysis was made using the integrated Harmony software (version 4.8, Perkin Elmer) [49].

### 2.7. Immunoprecipitation and GST Pull-Down Assay

Immunoprecipitation (IP) was performed as previously described in [43]. Briefly, cells were sonicated and centrifuged, and the resulting supernatant was precleared using Protein G Sepharose. Recombinant proteins were immunoprecipitated using a c-myc-specific antibody. For GST, pull-down assay *Escherichia coli* (*E. coli*) BL21 (DE3) cells were transformed with pGEX-4T-2-TSP1^1–221^, pGEX-4T-2-TSP1^1–221^ S93A, and pGEX-4T-2-TSP1^1–221^ S93D plasmids constructed earlier in our lab [40]. Transformed cells were cultured at 37 °C with shaking at 180 rpm until OD_600_ = 0.5. Protein expression was induced by adding 0.1 mM IPTG, followed by overnight incubation at 13 °C, as previously described in [40].

### 2.8. ELISA

TSP1 or IL-6 levels in the cell culture supernatant were quantified using a Human TSP1 ELISA Kit from Elabscience Biotechnology (E-EL-H1589) or a Mouse IL-6 ELISA Kit from Fine Test (EM0121), respectively. Absorbance was measured at 450 nm using Thermo Labsystems MULTISKAN GO microplate reader (Walthman, MA, USA), according to the manufacturer’s instructions.

### 2.9. MTT Assay

BPAECs or MOVAS cell proliferation was determined using MTT (3-[4,5-dimethylthiazolyl-2]-2,5-diphenyltetrazolium bromide) assay. Cells were seeded into flat-bottomed 96-well microtiter plates and treated with conditioned media (containing 10% FBS) of TSP1-transfected BPAEC. At the indicated time points, 10 μL of MTT solution (5 mg/mL) was added to each well and incubated at 37 °C for 1 h. Formazan crystals were dissolved in DMSO, and absorbance was measured at 540 nm using an automated plate reader (Thermo Labsystems MULTISKAN GO, Walthman, MA, USA).

### 2.10. Statistical Analysis

Statistical analyses were conducted using GraphPad Prism (version 9.5.1) with tests specified in figure legends. Differences were considered statistically significant at *p* < 0.05 (*), *p* < 0.01 (**), *p* < 0.001 (***), and *p* < 0.0001 (****). Densitometric analysis of immunoblots was carried out using ImageJ software (version 1.53a).

## 3. Results

### 3.1. TSP1^S93D^ Acts as a Negative Regulator of EC Migration

**Figure 1:**
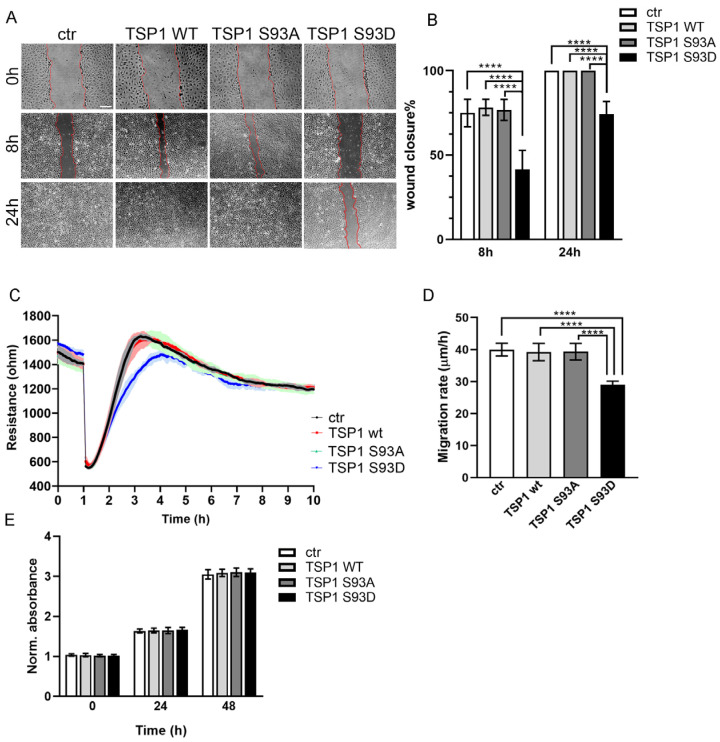
TSP1^S93D^ inhibits EC migration but not proliferation. (**A**) Representative images from a wound healing scratch assay using control, wild-type TSP1, and phosphomutant TSP1-transfected BPAECs. Scratches were made in a confluent EC monolayer 24 h post-transfection. Images were captured at 0, 8, and 24 h post-scratch. Scale bar = 100 µm. (**B**) Wound closure was evaluated by measuring the open area at each time point, normalized to the 0 h image (**** *p* < 0.0001). Statistical analysis was completed using one-way ANOVA followed by Tukey’s post hoc test. Data are presented as mean ± SD (*n* = 5). (**C**) Representative measurement of an in vitro wound healing assay performed using ECIS. Wounding was applied at 1 h. Each line represents the mean of three replicates ± SD. (**D**) Statistical analysis of EC migration rate was performed using one-way ANOVA with Tukey’s test (**** *p* < 0.0001). Data are represented as mean ± S.D (*n* = 8). (**E**) BPAEC were either untransfected (ctr) or transfected with TSP1^WT^-, TSP1^S93A^-, or TSP1^S93D^-expressing constructs. Cell proliferation was measured by an MTT assay. Absorbance values were normalized to 0 h. No significant differences in proliferation were observed between groups. Data represent SD (*n* = 6).

We showed earlier that TSP1^S93D^ inhibits in vitro tube formation of ECs and affects endothelial 3D spheroid formation. Since EC migration is essential for the formation of new blood vessels, we aimed to investigate the impact of TSP1S93 mutants on EC migration. BPAECs were transfected with pcDNA3.1myc-His TSP1 wt, -S93A, and -S93D plasmids, producing tagged TSP1 proteins for specific detection, with the myc-His tag lacking any c-myc-like functional activity. First, we performed a scratch assay on the confluent monolayer of EC 24 h post-transfection, and images were captured at various time points (Figure 1A). The reduced migratory capacity of TSP1^S93D^-expressing cells was already significant at 8 h post-wounding (Figure 1B). By 24 h, both control cells and those expressing TSP1^WT^ or the TSP1^S93A^ had successfully closed the wound, whereas TSP1^S93D^-expressing cells failed to do so, maintaining a larger open wound area. Next, the ECIS assay was used to monitor cell migration in real-time. ECs were seeded onto 8W10E electrodes and transfected with pcDNA3.1myc-His TSP1 plasmids. A defined electrical wound was applied 24 h post-transfection, while impedance was recorded continuously (Figure 1C). Consistent with our scratch assay findings, the ECIS assay showed that ECs expressing TSP1^S93D^ had a significantly slower migration rate compared to control cells and TSP1^WT^- or TSP1^S93A^-expressing cells (Figure 1D). Importantly, proliferation assessed over 48 h was unaffected by TSP1 overexpression, confirming that the impaired wound closure and reduced migration were due to altered migratory capacity rather than differences in proliferation (Figure 1E). These results suggest that the phosphorylation-mimicking S93D mutation of TSP1 has an anti-migratory effect independent of proliferation.

### 3.2. TSP1^S93D^ Inhibits FAK Phosphorylation During Cell Migration

Since TSP1^S93D^ strongly reduced endothelial migration, we hypothesize that the reduced motility of TSP1^S93D^-expressing cells might result from an inability to properly activate one or more signaling pathway during migration. The involvement of TSP1 linked to cell migration, such as protein kinase B (Akt), extracellular signal-regulated kinase 1/2 (ERK1/2), p38 mitogen-activated protein kinase (p38), and focal adhesion kinase (FAK) has been demonstrated in various cell types and experimental contexts [25,26,27]. To address this, we examined Akt, p38, ERK1/2, and FAK protein levels and their phosphorylation using phospho-specific antibodies in control and TSP1-transfected endothelial cells (Figure S1A). Our results showed that the overexpression of TSP1 did not alter the total protein levels or phosphorylation status of Akt, ERK1/2, p38, or FAK under basal conditions (Figure S1B).

**Figure 2:**
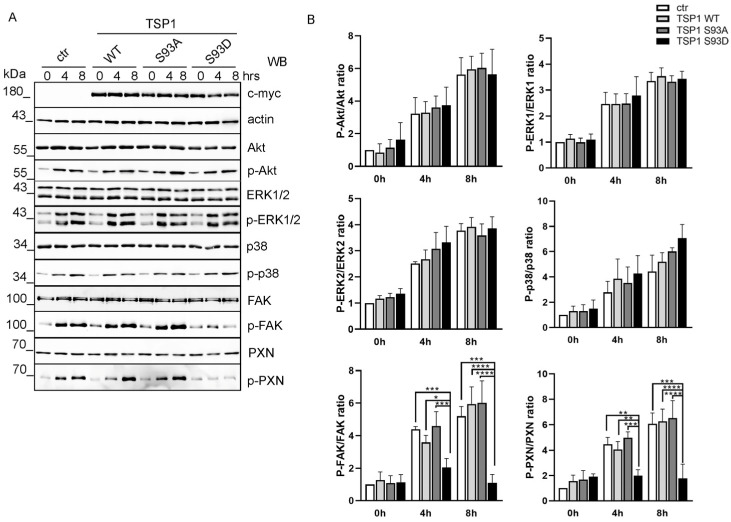
TSP1^S93D^ inhibits FAK signaling and downstream targets during cell migration. (**A**) Control or TSP1-transfected confluent monolayers of BPAECs were scratched 24 h post-transfection. Samples were collected at the indicated time points (0, 4, and 8 h). Overexpression of TSP1 and the levels of signaling protein were analyzed by Western blot. (**B**) Quantitative analysis was performed by densitometry of the Western blot bands. Statistical analysis was performed using one-way ANOVA followed by Tukey’s post hoc test (*n* = 3–5) (* *p* < 0.05, ** *p* < 0.01, *** *p* < 0.001, and **** *p* < 0.0001).

As these signaling pathways are dynamically regulated during active cell migration, we assumed that TSP1 might influence their activation during the migratory process. To study this, we transfected the ECs and performed a scratch assay to initiate cell migration. Cell lysates were collected at 0-, 4-, and 8-h time points after the wound was created. Phosphorylation dynamics were analyzed via Western blot (Figure 2A). We observed time-dependent phosphorylation of each pathway analyzed. In BPAECs, the Akt, ERK1/2, and p38 pathways were not affected by TSP1 overexpression at different time points compared to control (Figure 2B). In the case of FAK phosphorylation, there was no significant change in cells expressing TSP1^WT^ or TSP1^S93A^ mutant compared to control. However, cells expressing the TSP1^S93D^ mutant exhibited a significantly lower level of phospho-FAK compared to control and TSP1^WT^- and TSP1^S93A^-expressing cells. To further investigate downstream effects on focal adhesion signaling, we examined paxillin (PXN) phosphorylation, a well-known target of FAK. In line with the decrease in phospho-FAK, Tyr-phosphorylation of PXN was markedly reduced in TSP1^S93D^-expressing cells compared with control, TSP1^WT^, and TSP1^S93A^ cells, suggesting impaired focal adhesion turnover.

### 3.3. TSP1^S93D^ Mutant Shows Enhanced Binding with Integrin β1

**Figure 3:**
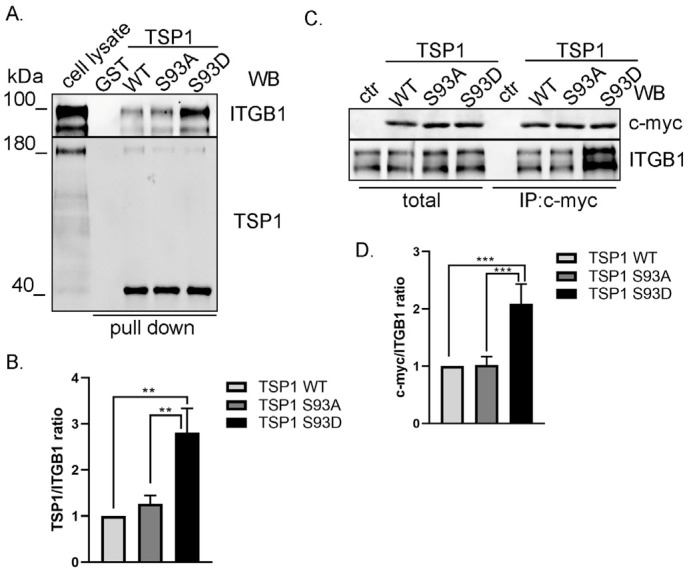
TSP1^S93D^ shows enhanced binding to ITGB1. (**A**) Bacterially expressed GST and GST-TSP1^1–221^WT, GST-TSP1^1–221^ S93A, and GST-TSP1^1–221^ S93D recombinant proteins immobilized on glutathione Sepharose beads were incubated with BPAEC lysate for pull-down assays. EC lysates and the eluted proteins were analyzed by Western blot using ITGB1- and TSP1-specific antibodies. (**B**) Quantitative analysis of pull-down samples. Statistical analysis was performed using one-way ANOVA (*n* = 4) (** *p* < 0.01). (**C**) Control and TSP1-transfected BPAECs were subjected to immunoprecipitation using c-myc antibody to purify recombinant TSP1 proteins. Total cell lysates and immunocomplexes were tested for c-myc and ITGB1 by Western blot. (**D**) Quantitative analysis of IP. Statistical analysis was performed using one-way ANOVA (*n* = 4) (*** *p* < 0.001).

Next, we intended to analyze upstream signaling of FAK to understand how TSP1^S93D^ inhibits FAK activation. TSP1 has been shown to interact with several integrins, including α3β1, α4β1, and α6β1. Integrins have been shown to bind to the N-terminal region of TSP1; therefore, a pull-down assay was performed using a glutathione S-transferase (GST)-tagged fragment of TSP1 comprising residues 1–221. GST, as a negative control, and purified GST-TSP1 proteins were immobilized on glutathione-agarose beads, and EC lysate was added. Interacting proteins were analyzed with TSP1- and integrin β1 (ITGB1)-specific antibodies (Figure 3A). TSP1 signals showed equivalent levels of each recombinant TSP1 protein. Moreover, all GST-TSP1 proteins (WT, S93A, and S93D) were able to bind to ITGB1, and no interaction was detected with GST alone, confirming the specificity of the observed binding. The TSP1^S93D^ fragment exhibited the strongest binding signal, suggesting enhanced affinity for ITGB1 relative to other TSP1 forms (Figure 3B). Full-length TSP1 tagged with c-myc was also immunoprecipitated from TSP1 overexpressing EC. IP complexes were analyzed using c-myc for recombinant TSP1- and ITGB1-specific antibodies (Figure 3C). The results showed the same trend as observed in our pull-down experiments. All TSP1 variants were able to bind ITGB1, with the TSP1^S93D^ mutant exhibiting the strongest binding (Figure 3D).

### 3.4. TSP1^S93D^ Alters ITGB1 Clustering

**Figure 4:**
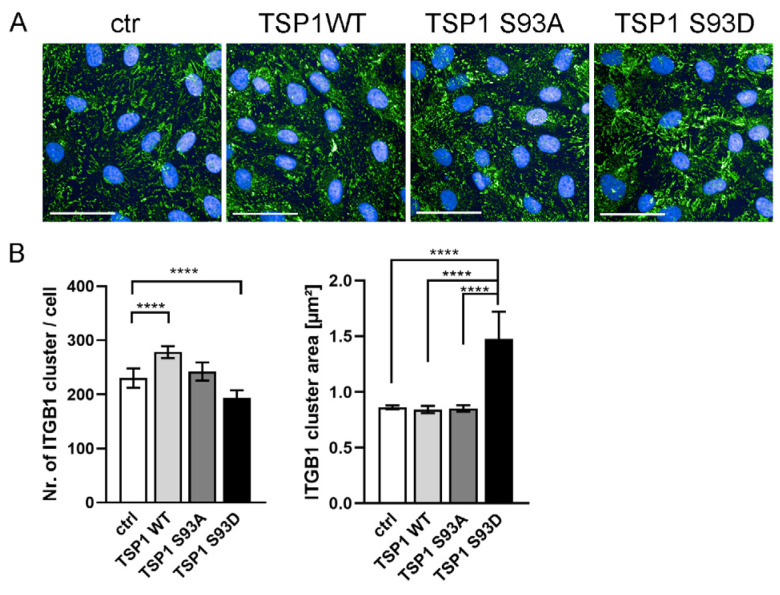
TSP1^S93D^ alters ITGB1 clusterization. (**A**) Representative images of control cells and cells expressing TSP1 recombinant variants, immunostained for ITGB1 (green) and nuclei (DAPI, blue). Images were acquired using the Opera Phenix HCS (PerkinElmer, Inc., Shelton, CT, USA. Scale bar: 100 μm. (**B**) Quantitative analysis of ITGB1 clusters was performed using the built-in Harmony software (version 4.8, Perkin Elmer). Statistical analysis was performed using one-way ANOVA. Data are presented as means ± S.D, and >10,000 cells were analyzed per condition. (**** *p* < 0.0001).

Given the established role of TSP1 in modulating integrin functions and extracellular matrix interactions, we asked whether expression of TSP1 and its phosphomutants affect the organization of ITGB1 clusterization. To examine whether TSP1 expression affects ITGB1 organization, cells overexpressing wild-type TSP1 or the S93A and S93D mutants were stained for ITGB1 and analyzed by high content screening (HCS) (Figure 4A). Overall staining patterns were similar in control cells and in cells expressing TSP1^WT^ or TSP1^S93A^. In contrast, cells expressing the phosphomimetic TSP1^S93D^ mutant showed visibly larger ITGB1 clusters. These differences were quantified using automated image analysis. ITGB1 clusters were identified, and the number of clusters per cell and their cluster area were compared (Figure 4B). We found a significant increase in ITGB1 cluster size in cells expressing TSP1^S93D^ compared to control cells, as well as cells expressing TSP1^WT^ or TSP1^S93A^. In parallel with this, the number of ITGB1 clusters was significantly lower due to TSP1^S93D^ overexpression.

### 3.5. Ser93 Side Chain Phospho-Modification Affects TSP1 Secretion

**Figure 5:**
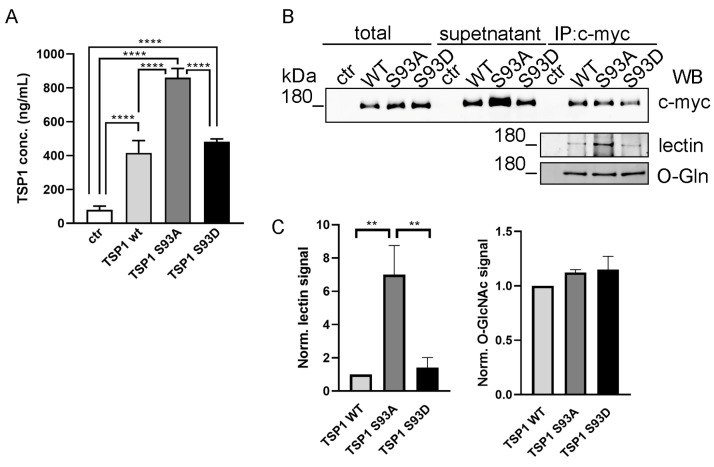
The S93A mutation of TSP1 increases its secretion via N-glycosylation. (**A**) Cell culture supernatants were analyzed for TSP1 levels using ELISA. Statistical analysis of TSP1 concentration was performed using one-way ANOVA followed by Tukey’s post hoc test (*n* = 7). Data are reported as means ± S.D. (**B**) TSP1 glycosylation was assessed by immunoprecipitating c-myc–tagged TSP1 proteins from the supernatant of transfected cells. Total cell lysates, supernatants, and IP complexes were tested for c-myc. IP complexes were further analyzed for glycosylation using lectin and O-GlcNac antibody by Western blot. (**C**) Quantification of glycosylation of immunoprecipitated recombinant TSP1 proteins. O-GlcNAc levels were not significantly different among groups. Data are shown as normalized signal intensity. (** *p* < 0.01 and **** *p* < 0.0001).

TSP1 is known as a secreted protein and undergoes extensive post-translational modifications, including glycosylation [50,51]. To investigate whether S93 residue influences TSP1 secretion, we used enzyme-linked immunosorbent assay (ELISA) to quantify the amount of TSP1 secreted into the culture medium by ECs transfected with TSP1 constructs. Overexpression of TSP1 increased the secreted TSP1 level compared to control cells. While TSP1^WT^ and TSP1^S93D^ mutants were secreted at similar levels, the TSP1^S93A^ mutant displayed a significantly higher amount (Figure 5A). This increase in secretion of the TSP1^S93A^ mutant suggested that Ser93 phosphorylation level may influence the glycosylation state of TSP1, thereby affecting its intracellular trafficking and release. Since the total TSP1 protein level (both endogenous and recombinant) can be measured by ELISA assay, culture supernatants from transfected ECs were subjected to immunoprecipitation (IP) using c-myc antibody to specifically examine only the recombinant proteins. Total cell lysates, supernatant samples, and IP complexes were analyzed by Western blot to assess glycosylation (Figure 5B). The secreted recombinant TSP1 proteins were detectable in the supernatants of the cells, with a stronger c-myc signal in the supernatant of cells expressing the TSP1^S93A^ mutant, supporting the observation of an enhanced secretion. During the IP, we set the used antibody concentration to be saturated with the recombinant TSP1; therefore, any difference in their modification would be comparable. According to this, c-myc signals were equal in the IP samples. To assess the glycosylation status of the immunoprecipitated TSP1, we tested the IP complexes using both O-linked β-N-acetylglucosamine (O-GlcNAc) antibody and lectin, which specifically recognizes N-acetylglucosamine and sialic acid residues on glycoproteins. Blots of the IP samples revealed a distinct signal corresponding to the molecular weight of recombinant TSP1, which was absent in control (non-transfected) samples. On the lectin blot, a significantly stronger signal was observed for TSP1^S93A^, suggesting an increased level of glycosylation on this mutant form of TSP1 (Figure 5C). The intensity of the signals detected by the O-GlcNAc antibody was comparable across samples.

### 3.6. TSP1^S93D^ Enhances SMC Proliferation and Migration

After confirming that all recombinant TSP1 variants are secreted by ECs, we intended to analyze how these variants might affect SMC behavior. TSP1 can bind to multiple receptors and proteins, not just on EC, but also on SMC [52]. To explore these functions, we used MOVAS cells as a model system, which is widely used to study SMC behavior [53,54]. Importantly, the TSP1 protein sequence is highly conserved across species, making MOVAS cells a relevant model to study the effects of recombinant TSP1 carrying site-specific mutations. We used EC to produce recombinant TSP1 variants and collected their complete conditioned media. As endothelial cells also secrete endogenous TSP1, the conditioned media contained both endogenous and recombinant proteins. Because the different TSP1 variants were secreted at varying levels, the conditioned media were diluted with complete media and normalize the final total TSP1 concentration to 300 ng/mL before treatment of MOVAS cells. This concentration was chosen to ensure sufficient receptor engagement while remaining within a biologically relevant range of TSP1 [55,56]. Under these conditions, endogenous TSP1 levels were comparable across samples, and any observed differences in cellular response were, therefore, attributed to the presence of the respective recombinant TSP1 variants rather than to differences in total TSP1 abundance.

**Figure 6:**
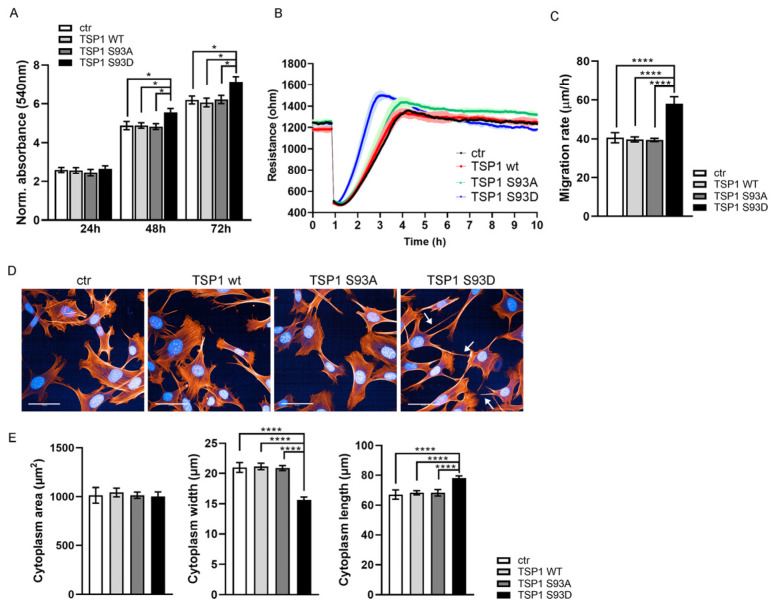
TSP1^S93D^ enhances SMC migration and proliferation and induces morphological changes. (**A**) MOVAS cells were seeded at 10% confluence and treated with conditioned media from non-transfected (ctr) or TSP1-transfected BPAECs. Proliferation was assessed using MTT, with absorbance measured at 540 nm at 24, 48, and 72 h. Data are shown as means ± SEM (*n* = 12). Statistical analysis was performed using one-way ANOVA with Tukey’s post hoc test (* *p* < 0.01). (**B**) Migration of treated MOVAS cells was measured using an ECIS-based wound healing assay. Data presented mean ± S.D. from three chambers per condition. (**C**) Statistical analysis of migration rates was performed using one-way ANOVA with Tukey’s post hoc test (*n* = 5; means ± S.D.; **** *p* < 0.0001). (**D**) Representative images of control and TSP1-treated MOVAS cells analyzed by HCS. Actin filaments were stained with Texas Red phalloidin (red) and nuclei with DAPI. White arrows indicate filopodia of cells. Scale bars: 50 μm. (**E**) Morphological parameters of MOVAS cells were analyzed using Harmony software on the Opera Phenix HCS system. Data are presented as mean ± SD (1000–1600 cells per well, *n* = 4). Statistical analysis was performed using ANOVA (**** *p* < 0.0001).

First, an MTT assay was performed on sub-confluent MOVAS cells to assess cell viability and proliferation (Figure 6A). It was found that media containing the TSP1^S93D^ mutant significantly enhanced MOVAS cell proliferation compared to the TSP1^WT^ and TSP1^S93A^ forms. Cell migration was measured using ECIS in vitro wound healing (Figure 6B). Interestingly, we found that MOVAS cells treated with TSP1^S93D^-containing conditioned media showed significantly faster cell migration than the control or TSP1^WT^- or TSP1^S93A^-treated cells (Figure 6C). We also tested whether there is any cytoskeletal phenotype change due to TSP1 treatment of MOVAS cells. Cells were plated at sub-confluent density to allow visualization of individual cells. After treatment with conditioned media from EC for 24 h, the actin cytoskeleton and nuclei were visualized using Texas Red phalloidin and DAPI staining, respectively (Figure 6D). Cell morphology was evaluated using Opera Phenix high content screening (HCS). No significant difference was found in the cytoplasm area of cells; however, cells treated with TSP1^S93D^-containing media displayed an elongated shape, characterized by a significant increase in cytoplasm length and a decrease in cytoplasm width (Figure 6E). Interestingly, on the confocal images, we observed a higher number of filopodia of TSP1^S93D^-treated cells, frequently associated with enhanced motility, that corresponds to our cell migration experiments.

### 3.7. TSP1^S93D^ Induces Phenotype Change in SMC from Contractile to Synthetic Type

**Figure 7:**
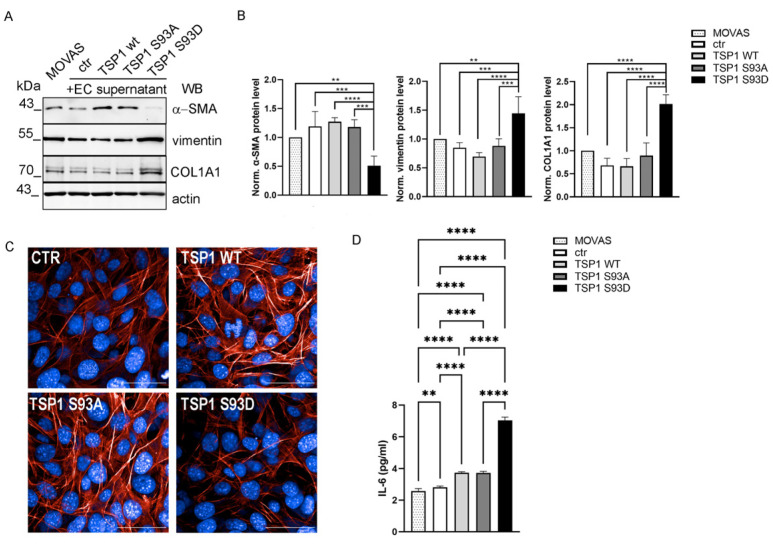
TSP1^S93D^ induces a shift toward a synthetic-like state in SMCs and increases IL-6 secretion. (**A**) MOVAS were treated with conditioned media from TSP1 overexpressing ECs. Expression levels of indicated proteins were tested by Western blot. Actin was used as a loading control. (**B**) Densitometric analysis of Western blot signals. Statistical analysis was performed using one-way ANOVA with Tukey’s test (*n* = 4). (**C**) Representative images of control and TSP1-treated MOVAS cells analyzed by HCS. Actin filaments were stained with Texas Red phalloidin (red) and nuclei with DAPI. Scale bars: 50 μm. (**D**) Conditioned media treated and untreated MOVAS cell supernatants were analyzed by ELISA for IL-6. Statistical analysis was performed using one-way ANOVA (*n* = 9). Data are reported as means ± S.D. (** *p* < 0.01, *** *p* < 0.001, and **** *p* < 0.0001).

Numerous reviews have established that SMCs undergo phenotypic switching from a contractile to a synthetic state, which contributes to their functional variety, making them a useful model system to study smooth muscle cell plasticity [57,58]. A contractile, “dormant” phenotype is characteristic of differentiated SMCs, and a synthetic phenotype is marked by dedifferentiation [59]. Differentiated SMCs, displaying a spindle-shaped morphology, express high levels of contractile proteins, including α-smooth muscle actin (α-SMA) [60]. In contrast, factors such as TGFβ, platelet-derived growth factor (PDGF)-BB, angiotensin II, or pathological conditions can induce a switch to a synthetic phenotype, wherein SMCs express reduced levels of contractile proteins and increased levels of proteins associated with proliferation, migration, fibrosis, and inflammation [61,62,63]. These cells also exhibit enhanced proliferative and migratory capacities, with an increased secretion of ECM proteins and pro-inflammatory cytokines [64]. Upon transitioning to myofibroblast-like phenotype, SMCs upregulating matrix protein expression, such as collagen I, II, and III (COL1A1-3) [65], are accompanied by the progressive loss of contractile markers [66]. Given that the increased motility and proliferation of cells are hallmarks of synthetic SMCs, our results suggested a shift in cell phenotype driven by the TSP1^S93D^. To analyze this phenotypic transition, the expression of molecular markers indicative of synthetic or contractile phenotype SMC was assessed. MOVAS cells were treated with conditioned media from EC for 72h, with the longer treatment period chosen to allow sufficient time for both gene expression and protein level changes to occur. We tested protein expression levels of α-SMA, vimentin, and COL1A1 by Western blot (Figure 7A). TSP1^S93D^ treatment displayed reduced α-SMA expression, alongside increased levels of vimentin and COL1A1, consistent with a transition toward a synthetic phenotype (Figure 7B). To further support these molecular findings, we examined actin organization by immunofluorescence staining using HCS. Well-defined stress fibers are typically seen in contractile SMCs, while their loss or disorganization is associated with the synthetic state [67]. As seen in Figure 7C, control cells display relatively thin, less bundled stress fibers with weaker organization, whereas TSP1^WT^- and TSP1^S93A^-treated cells exhibit strong, thick, and well-organized stress fibers spanning across the cytoplasm, which are absent in TSP1^S93D^-treated cells. Synthetic SMCs are usually involved in tissue remodeling, cell proliferation, or inflammation, and they secrete different pro-inflammatory cytokines, such as IL-6, which is often used as a marker of vascular injury response. Next, we measured IL-6 concentration of MOVAS cells by ELISA (Figure 7D). We found a slightly elevated concentration of IL-6 in MOVAS cells treated with the EC supernatant, compared to the untreated cells. TSP1 treatment increased IL-6 secretion in all cases, with the highest concentration in TSP1^S93D^-treated cells, which was significantly higher than control or TSP1^WT^- or TSP1^S93A^-treated cells.

## 4. Discussion

TSP1 exhibits controversial and context-dependent behavior in ECs and SMCs. In ECs, TSP1 typically acts as an anti-angiogenic factor by inhibiting NO signaling and inducing apoptosis [19], whereas in smooth muscle cells, it can promote proliferation and migration, contributing to vascular remodeling. However, these divergent effects are strongly influenced by the structural complexity of TSP1, specifically its multiple functional domains that interact with various receptors like CD36, CD47, and integrins [16,17,19]. Peptides derived from TSP1, such as those mimicking its anti-angiogenic sequences, are being explored therapeutically to modulate its effects selectively [68,69,70]. This structure- and domain-specific activity highlights just how versatile TSP1 is in vascular biology. TSP1 is well known for its role in regulating angiogenesis, ECM remodeling, and cell-matrix interactions, but insight into how post-translational modifications like phosphorylation influence its cellular effects has remained limited. Our previous study highlighted the significance of the reversible phosphorylation of TSP1 at Ser93 by PKC and PP2A-B55α holoenzymes in EC, showing that TSP1^S93D^-expressing cells formed smaller spheroids and exhibited reduced capacity for tube formation [40]. To better understand the underlying molecular mechanism behind these differences, we focused on EC migration, one of the crucial steps during angiogenesis. TSP1^S93D^ phosphomimetic mutant overexpressing cells showed decreased migratory rate and slower wound closure compared to control and TSP1^WT^- or TSP1^S93A^-expressing cells. TSP1 can modulate FAK activity through interactions with integrins such as α3β1 [71] and β1 [72], key proteins for cell adhesion to the ECM. However, the interaction between TSP1 and FAK is domain specific. The N-terminal domain of TSP1 can promote cell adhesion and spreading, leading to the activation of FAK [73]. In contrast, the type I repeats of TSP1 have been shown to inhibit FAK phosphorylation, possibly through interactions with CD36 or by affecting the integrin signaling [74]. We showed that, during active cell migration, elevated phospho-FAK level was found in control and TSP1^WT^- and TSP1^S93A^-expressing cells, but TSP1^S93D^ showed an inhibitory effect. FAK regulates the dynamics of adhesions, and its activation combines with Src family kinases, which starts downstream signaling pathways by phosphorylating other proteins to control various cellular processes [75]. PXN, a member of the LIM-domain protein family, functions as a structural scaffold and signaling hub protein in focal adhesions. It is one of the most critical proteins for the formation and function of integrin-mediated adhesions. Its interactions with binding partners are regulated by its phosphorylation status [76]. Specifically, tyrosine phosphorylation at Y31 and Y118, mediated by the FAK-Src complex, recruits both activators and inhibitors for Rho-GTPases [77,78]. The lack of FAK phosphorylation in TSP1^S93D^-expressing cells ultimately resulted in the lack of PXN phosphorylation. It was reported that TSP1 interacts with various integrins, including αvβ3, αIIbβ3, α3β1, α4β1, and α5β1, which mediate many of its biological functions [9]. Although integrin-mediated cell adhesion and activation can occur in the absence of PXN, cell spreading and proliferation depend on PXN recruitment to these sites [79,80]. Several studies have further identified specific integrins as functional receptors for TSP1 in the context of angiogenesis. For example, α3β1 integrin has been shown to modulate EC behavior and angiogenesis in vitro [71], while α4β1 integrin influences angiogenesis in vivo and regulates EC responses to TSP1 in vitro [81]. The anti-migratory effect of TSP1 is either mediated via CD36 [22] or regulated by binding to ITGB1 [72]. Consistent with previous reports, we found that TSP1 interacts with ITGB1; however, the S93D mutation altered ITGB1 cluster formation. Our results suggest that enhanced binding of the TSP1^S93D^ to ITGB1 interferes with FAK activation, leading to reduced PXN phosphorylation and, consequently, impaired cell migration.

TSP1 is secreted not only to act on ECs but also on other cell types, such as SMCs. Therefore, we investigated how Ser93 mutations affect its secretion. TSP1 is heavily glycosylated, with several unique modifications. Its TSR domains undergo O-fucosylation and C-mannosylation [50]. N-glycosylation has been identified at residues N248, N360, N708, and N1067 [51], and O-GalNac modifications occur at T289, T292, T293, T302, and T638 [82]. In addition, W368, W420, W423, and W480 are C-mannosylated, while S377, T432, and T489 carry O-linked disaccharides [50]. O-glycosylation supports its proper folding and facilitates transport from the endoplasmic reticulum [83,84]. Meanwhile, the extensive N-linked modifications have been studied in relation to clearance and uptake of TSP1 [85], glycosaminoglycan binding, and its anti-adhesive activity [86]. We found that the higher secretion of TSP1^S93A^ phosphonull mutant correlated with its increased N-linked glycosylation, but no difference was found in its O-glycosylation. Although none of the known glycosylation sites are near the Ser93 side chain, there may still be unidentified ones. Phosphorylation could still indirectly influence the accessibility of glycosylation sites, thereby affecting the dynamics of its secretion. While the full 3D structure of TSP1 remains unsolved, the structure of TSP1 N-terminal domain in complex with synthetic pentameric heparin has been determined [87]. The crystal structure of TSP1 N-terminal reveals a β-sandwich composed of 13 antiparallel β-stands and 6 α-helices [87]. Ser93 is in the β5 stand, near several amino acids classified as major heparin-binding sites.

As the regulation of blood vessel growth and function is influenced by interactions between EC and SMC within the vessel wall [5], we investigated how the presence of the different secreted TSP1 phosphomutants affects SMC behavior. In contrast to their effects on ECs, TSP1^S93D^ promoted proliferation and migration of SMCs. It should be considered that the EC culture media used to treat MOVAS cells contained not only recombinant TSP1 but also various growth factors and ECM components. However, the TSP1^S93D^-containing culture media induced changes in SMC phenotype, as enhanced filopodia formation and cell elongation were observed, suggesting a phenotypic shift toward a synthetic and proliferative state. Although differentiated cells are capable of trans-differentiation, previous studies have emphasized the inherent complexity of SMC heterogeneity, both in terms of origin and the spatiotemporal variation in expression of differentiation markers [88]. Our results indicate that TSP1^S93D^ induces an SMC phenotypic switch toward a synthetic phenotype, as evidenced by increased expression of COL1A1 and vimentin, alongside a reduction in α-SMA levels. Synthetic SMCs also secrete a range of cytokines and chemokines, such as IL-6, monocyte chemoattractant protein-1 (MCP-1), IL-1β, tumor necrosis factor-α (TNF-α), and chemotactic factors, which contribute to oxidative stress and vascular inflammation [89]. In our study, TSP1 treatment led to a significant increase in IL-6 secretion, especially for TSP1^S93D^. Growing evidence supports a role for IL-6 in regulating SMC migration [90,91]. IL-6 has been shown to induce actin cytoskeleton reorganization and promote FAK phosphorylation, suggesting its function as a chemoattractant that enhances SMC motility. This activity plays a major role for IL-6 in the pathogenesis of arterial stenotic disease, in line with its elevated levels in atherosclerosis and restenosis [92,93].

Atherosclerosis, the primary driver of cardiovascular disease, develops through a complex sequence of histological changes in the arterial wall, primarily driven by chronic inflammation and abnormalities in lipid metabolism. In early stages (pro-atherosclerosis), ECs are damaged and activated, leading to increased vascular permeability and the release of adhesion molecules, cytokines, and chemokines [94]. The phenotypic plasticity of VSMCs is a critical component of atherosclerosis progression. Under physiological conditions, VSMCs maintain a quiescent, contractile phenotype that contributes to vessel tone and integrity. However, in response to inflammatory stimuli and matrix signals, such as elevated TSP1, VSMCs can undergo dedifferentiation into a synthetic phenotype characterized by increased proliferation, migration, and extracellular matrix production [67]. This shift contributes to intimal thickening, plaque growth, and fibrosis within atherosclerotic lesions. Phenotypic switching of SMCs has been implicated in the pathogenesis of several vascular diseases, such as aneurysms, hypertension, mechanical injury, and atherosclerosis as well [59,62,95,96]. We showed that the phosphomimetic TSP1^S93D^ mutant appeared to enhance these early atherogenic events, promoting inflammatory signaling and disrupting EC-SMC communication. These findings suggest that TSP1, through its phosphorylation status, may act as a mediator of vascular cell cross-talk and contribute to early atherosclerotic remodeling.

## 5. Conclusions

In conclusion, our data provide a more granular view of how specific molecular modifications influence TSP1 function. Ser93 phosphorylation emerges as a pivotal switch that alters TSP1 behavior, suppressing EC migration and angiogenic potential while promoting SMC activation and phenotypic switching. These divergent effects underscore the complexity of TSP1 signaling in the vascular microenvironment and suggest that targeting specific TSP1 modifications or their downstream signaling consequences could be a novel strategy for modulating angiogenesis or vascular remodeling in disease contexts. While these observations were made in vitro, they provide a useful starting point for future in vivo studies to examine whether Ser93-dependent TSP1 signaling plays a similar role under physiological and pathological conditions and for exploring phosphomimetic or peptide-based therapeutic approaches.

## Figures and Tables

**Table 1:** Antibodies utilized in Western blot.

Antibody	Dilution	Vendor (Cat#)
actin (20-33)	1:1000	Sigma (St. Louis, MO, USA) (A5060)
Akt1 (C73H10)	1:1000	Cell Signaling Technologies (Danvers, MA, USA) (2938S)
anti-mouse IgG HRP-linked	1:5000	Cell Signaling Technologies (Danvers, MA, USA) (#7076S)
anti-rabbit IgG HRP-linked	1:5000	Cell Signaling Technologies (Danvers, MA, USA) (#7074S)
c-myc (9E10)	1:500	Invitrogen (Carlsbad, CA, USA) (13-2500)
COL1A1 (3G3)	1:500	Santa Cruz Biotechnology (Dallas, TX, USA) (sc-293182)
ERK 1/2 (C-9)	1:1000	Santa Cruz Biotechnology (Dallas, TX, USA) (sc-514302)
FAK (H-1)	1:500	Santa Cruz Biotechnology (Dallas, TX, USA) (sc-1688)
integrin β1 (A-4)	1:1000	Santa Cruz Biotechnology (Dallas, TX, USA) (sc-374429)
O-GlcNac (CTD110.6)	1:1000	Cell Signaling Technologies (Danvers, MA, USA) (9875S)
p38 alpha/beta MAPK (A-12)	1:1000	Santa Cruz Biotechnology (Dallas, TX, USA) (sc-7972)
p-Akt (S473)	1:1000	Cell Signaling Technologies (Danvers, MA, USA) (4060S)
paxillin (B-2)	1:1000	Santa Cruz Biotechnology (Dallas, TX, USA) (sc-365379)
p-ERK (E-4)(Tyr204/Tyr187)	1:1000	Santa Cruz Biotechnology (Dallas, TX, USA) (sc-7383)
p-FAK (Tyr397)	1:1000	Santa Cruz Biotechnology (Dallas, TX, USA) (sc-81493)
p-p38 MAPK (E-1) (Tyr182)	1:1000	Santa Cruz Biotechnology (Dallas, TX, USA) (sc-166182)
p-paxillin (A-5)	1:1000	Santa Cruz Biotechnology (Dallas, TX, USA) (sc-365020)
thrombospondin-1 (C-8)	1:1000	Santa Cruz Biotechnology (Dallas, TX, USA) (sc-393504)
vimentin (R28)	1:1000	Cell Signaling Technologies (Danvers, MA, USA) (3932S)
α-SMA	1:1000	R&D Systems Bio-techne (Minneapolis, MN, USA) (MAB1420)

## Data Availability

The data that support the findings of this study are available from the corresponding author upon reasonable request.

## Supplementary Materials

The following supporting information can be downloaded at: https://www.mdpi.com/article/10.3390/biom16010084/s1, Figure S1. Overexpression of TSP1 does not alter the expression or baseline phosphorylation of key signaling proteins. File S1. Original Western blot images.

## Abbreviations

The following abbreviations are used in this manuscript: Akt: protein kinase B; α-SMA: α-smooth muscle actin; BPAEC: bovine pulmonary artery endothelial cells; cGMP: cyclic guanosine monophosphate; COL1A1: collagen type I alpha 1 chain; DAPI: 4′,6-diamidino-2-phenylindole; EC: endothelial cell; ECIS: Electric cell-substrate impedance sensing; ECM: extracellular matrix; ELISA: enzyme-linked immunosorbent assay; ERK1/2: Extracellular Signal-Regulated Kinases ½; FAK: Focal Adhesion Kinase; GST: glutathione S-transferase; HCS: High-Content Screening; IP: immunoprecipitation; ITGB1: integrin β1; LRP1: low-density lipoprotein receptor-related protein 1; MAPK/p38: p38 mitogen-activated protein kinase; MCP-1: monocyte chemoattractant protein-1; MOVAS: mouse aortic vascular smooth muscle cells; MTT: 3-[4,5-dimethylthiazolyl-2]-2,5-diphenyltetrazolium bromide; NF-κB: nuclear factor kappa B; O-GlcNAc: O-linked β-N-acetylglucosamine; PC: procollagen; PDGF-BB: platelet-derived growth factor-BB; PI3K: phosphoinositide-3-kinase; PKC: protein kinase C; PP2A: protein phosphatase 2A; PXN: paxillin; SMC: smooth muscle cell; TGF-β: transforming growth factor-beta; TLR4: Toll-like receptor 4; TNF-α: tumor necrosis factor-α; TSP: thrombospondin; TSR: thrombospondin-type repeat; VEGF: vascular endothelial growth factor; VEGFR: vascular endothelial growth factor receptor; VLDLR: very low-density lipoprotein receptor; VSMC: vascular smooth muscle cell; vWC: von Willebrand type C.
