# Supplementary material for: Phosphomimetic Thrombospondin-1 Modulates Integrin β1-FAK Signaling and Vascular Cell Functions
Source: Biomolecules. 2026 Jan 4;16(1):84. doi: 10.3390/biom16010084 (PMC12839108; doi:10.3390/biom16010084)
Supplement: Supplementary file 1 [file biomolecules-16-00084-s001.zip › Figure S1.pdf]

Suppl. figures:

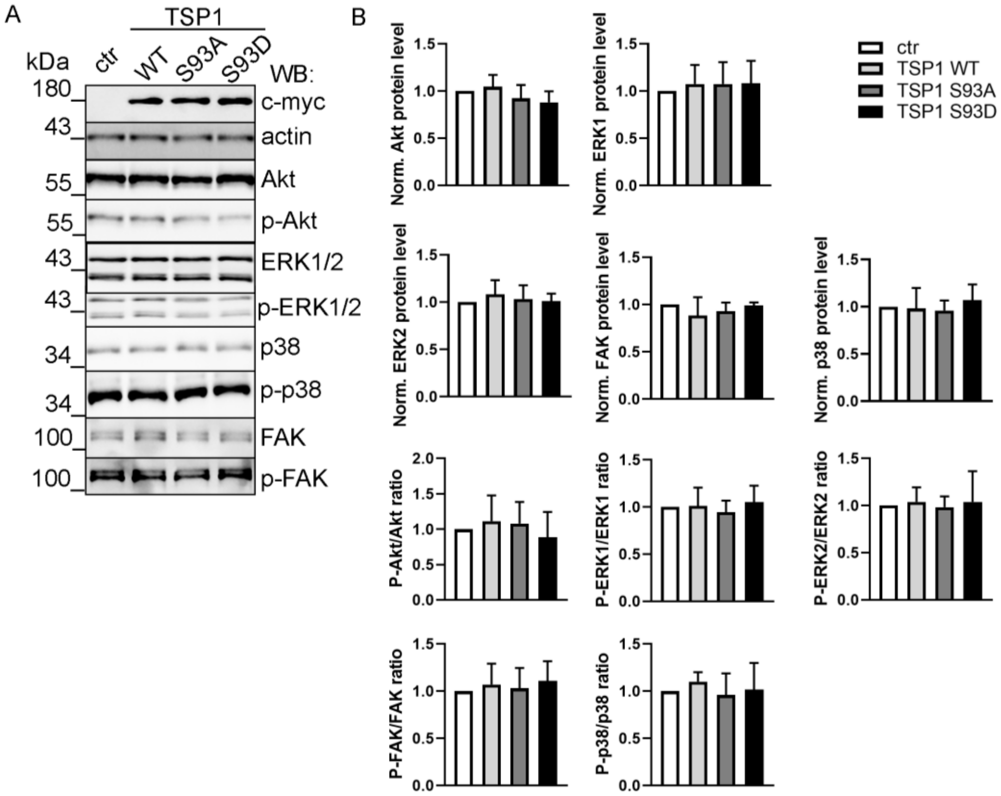

Supplementary figure S1. Overexpression of TSP1 does not alter the expression or baseline phosphorylation of key signaling proteins (A) BPAECs were transfected with pcDNA myc-His TSP1^{WT}, TSP1^{S93A} and TSP1^{S93D} plasmids. Protein expression was analyzed 24h post-transfection. Overexpression of different TSP1 proteins was confirmed using c-myc antibody. Expression and phosphorylation levels of signaling proteins were analyzed by Western blot. Actin was used as a loading control. (B) Densitometric analysis of Western blot signals is shown. No significant differences in protein levels or phosphorylation levels were observed between groups.
