# Supplementary material for: Phosphomimetic Thrombospondin-1 Modulates Integrin β1-FAK Signaling and Vascular Cell Functions
Source: Biomolecules. 2026 Jan 4;16(1):84. doi: 10.3390/biom16010084 (PMC12839108; doi:10.3390/biom16010084)
Supplement: Supplementary file 1 [file biomolecules-16-00084-s001.zip › File S1. original images.pdf]

Phosphomimetic thrombospondin-1 modulates integrin β1-FAK signaling and vascular cell functions

Assala Raya [1] , Bálint Bécsi [1] and Anita Boratkó [1,*]

[1]Department of Medical Chemistry, Faculty of Medicine, University of Debrecen, Egyetem tér 1,
H-4032 Debrecen, Hungary;

*Correspondence: boratko@med.unideb.hu; Tel.: (+36-52-412-345)

Figure 2A.

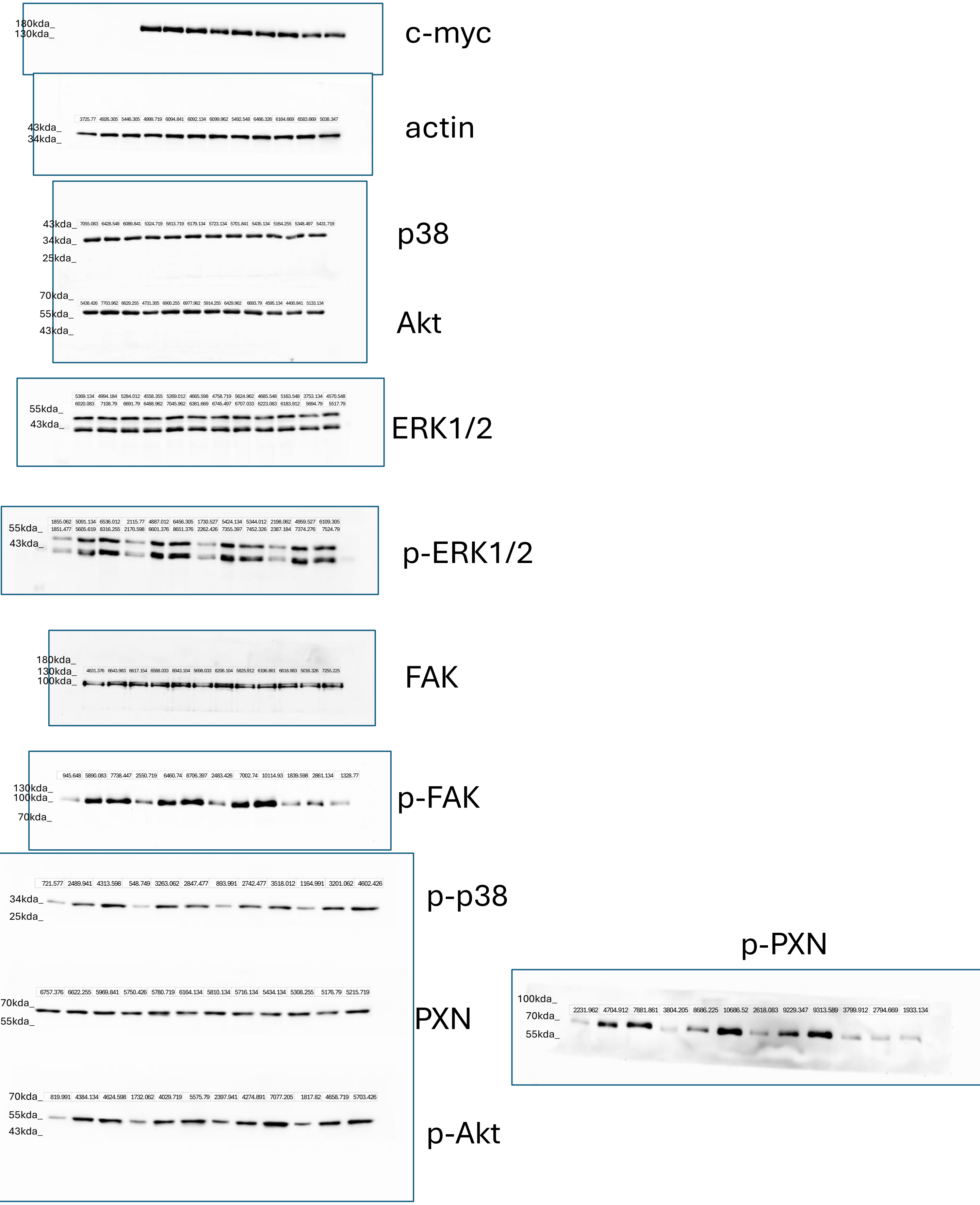
c-myc
180kda_
130kda_
actin
43kda_
34kda_
p38
43kda_
34kda_
25kda_
Akt
70kda_
55kda_
43kda_
ERK1/2
55kda_
43kda_
p-ERK1/2
55kda_
43kda_
FAK
180kda_
130kda_
100kda_
p-FAK
130kda_
100kda_
70kda_
p-p38
34kda_
25kda_
PXN
70kda_
55kda_
p-Akt
70kda_
55kda_
43kda_
p-PXN
100kda_
70kda_
55kda_

Figure 3A.

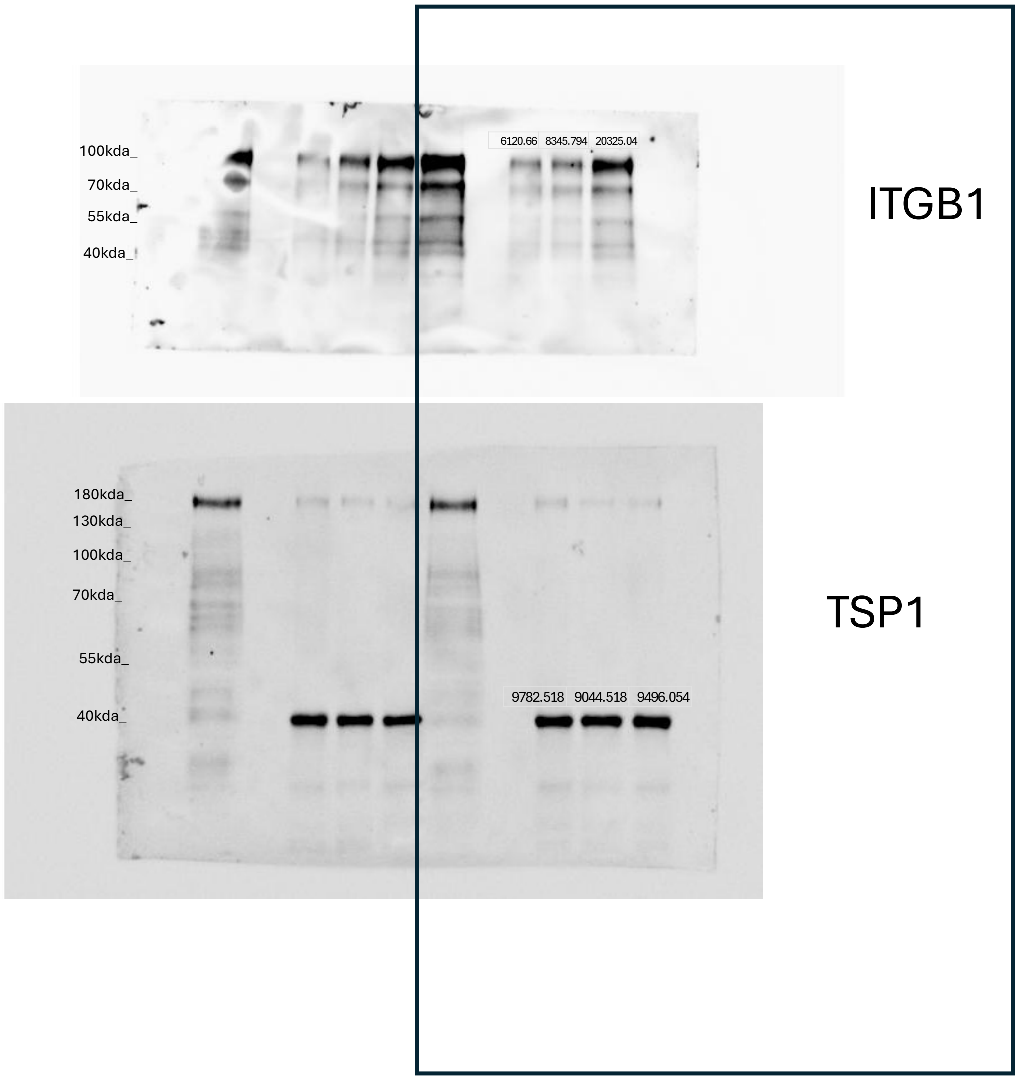
100kda_
70kda_
55kda_
40kda_
6120.66
8345.794
20325.04
ITGB1
180kda_
130kda_
100kda_
70kda_
55kda_
40kda_
9782.518
9044.518
9496.054
TSP1

Figure 3C.

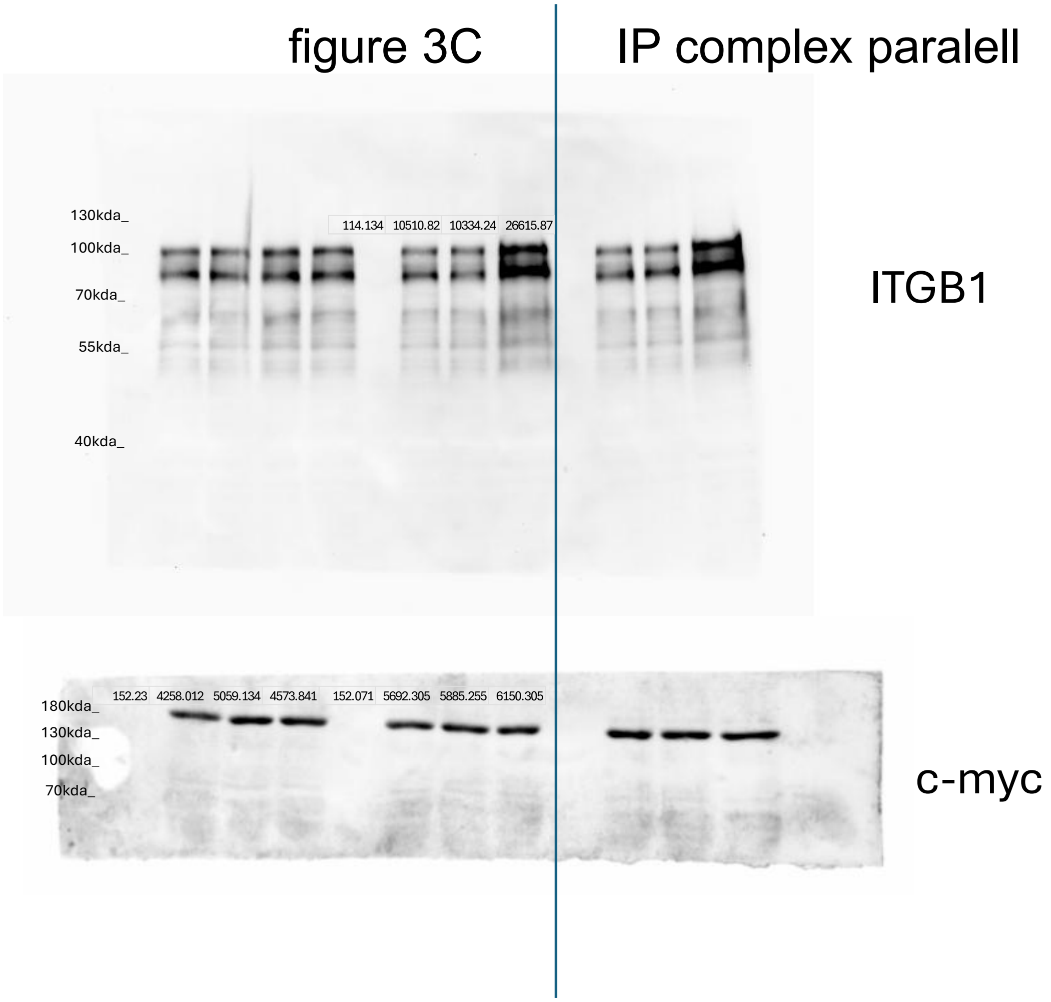

figure 3C
IP complex paralell
130kda_
100kda_
70kda_
55kda_
40kda_
114.134
10510.82
10334.24
26615.87
ITGB1
180kda_
130kda_
100kda_
70kda_
152.23
4258.012
5059.134
4573.841
152.071
5692.305
5885.255
6150.305
c-myc

Figure 5B.

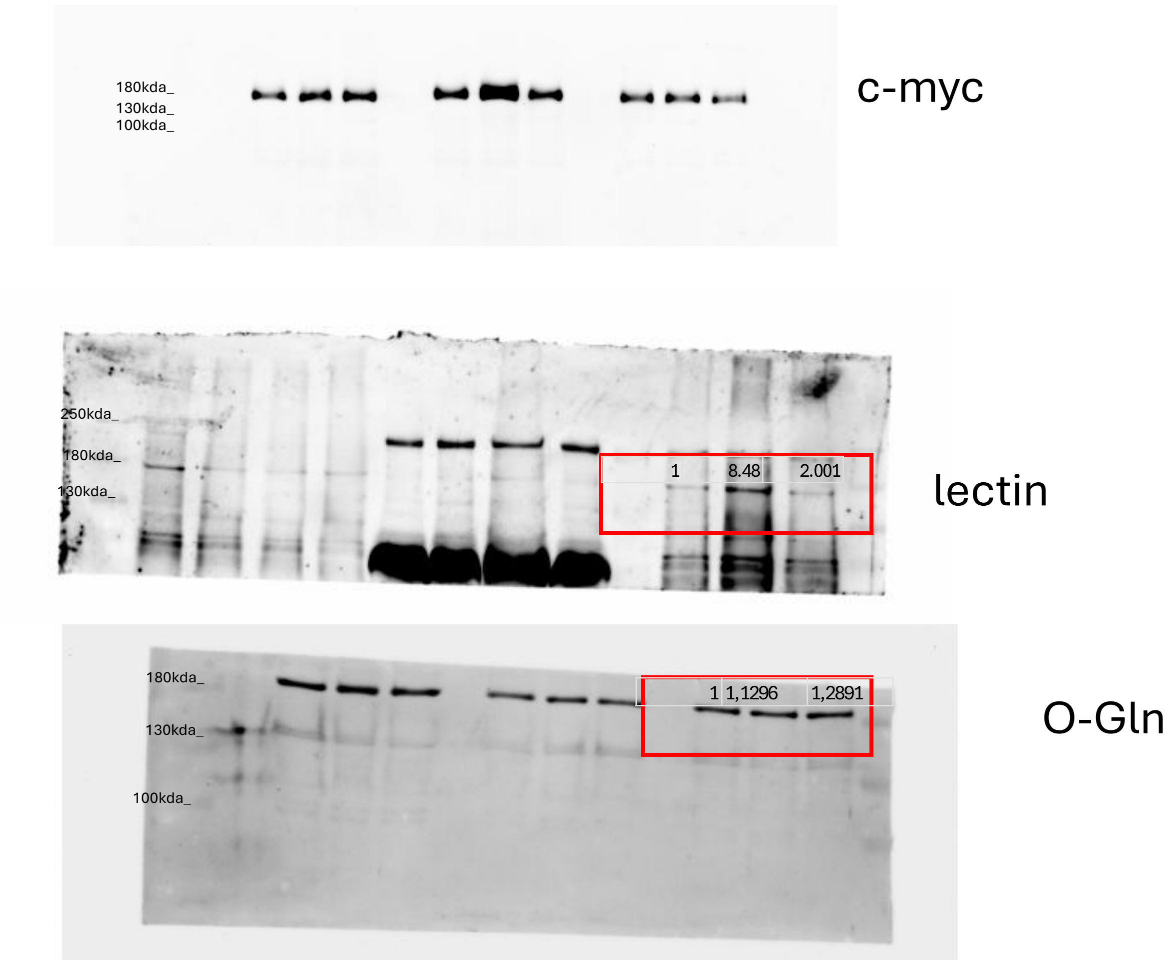
180kda_
130kda_
100kda_
c-myc
250kda_
180kda_
130kda_
1
8.48
2.001
lectin
180kda_
130kda_
100kda_
1
1,1296
1,2891
O-Gln

Figure 7A.

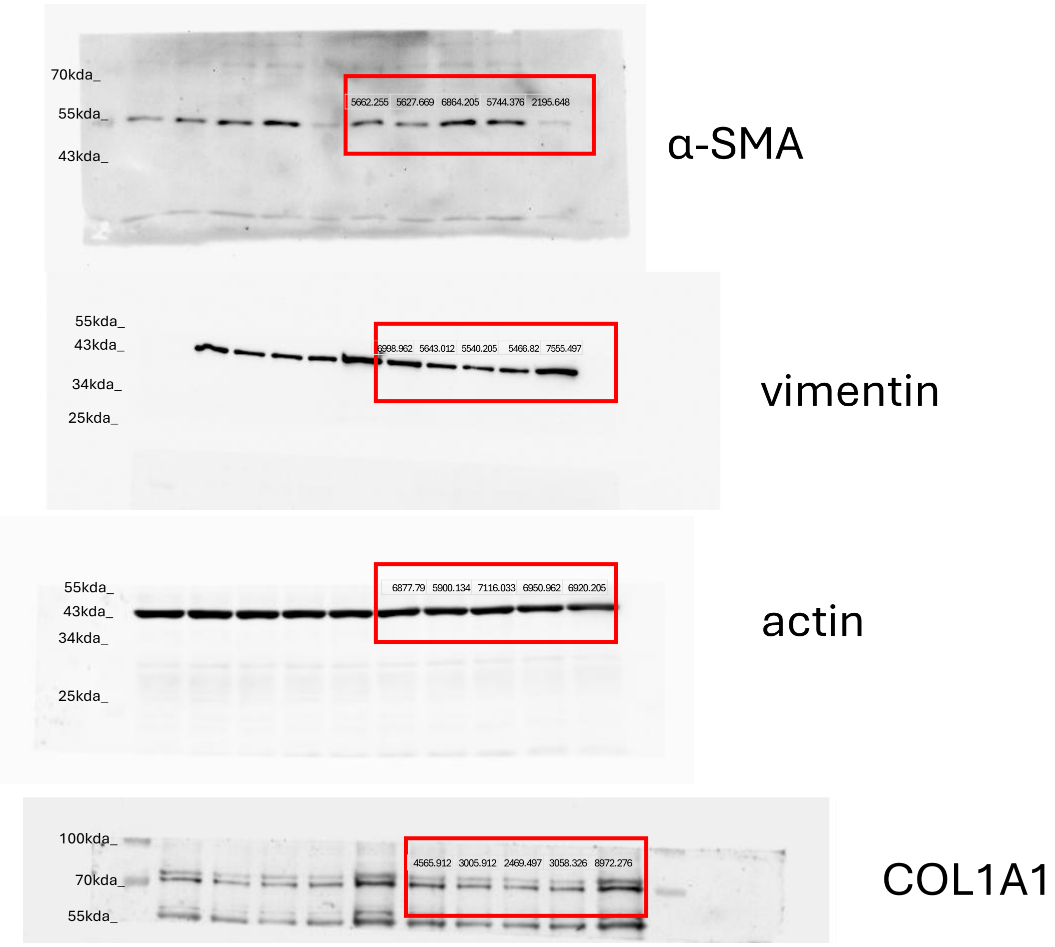
70kda_
55kda_
43kda_
5662.255 5627.669 6864.205 5744.376 2195.648
α-SMA
55kda_
43kda_
34kda_
25kda_
6998.962 5643.012 5540.205 5466.82 7555.497
vimentin
55kda_
43kda_
34kda_
25kda_
6877.79 5900.134 7116.033 6950.962 6920.205
actin
100kda_
70kda_
55kda_
4565.912 3005.912 2469.497 3058.326 8972.276
COL1A1
